# Neuromonitoring of perioperative microembolization and its association with postoperative stroke after carotid endarterectomy

**DOI:** 10.1007/s00423-026-04324-8

**Published:** 2026-10-09

**Authors:** Mohammed Al-Falahi, Jelle Frankort, Gabriel Schima, Bernhard Hruschka, Quentin Cappel, Werner Mess, Christian Uhl, Alexander Gombert, Panagiotis Doukas

**Affiliations:** 1 Department of Vascular Surgery, Medical Faculty, RWTH Aachen University, Aachen, Germany; 2 Department of Vascular Surgery, MUMC+, Maastricht, The Netherlands; 3 Department of Clinical Neurophysiology, MUMC +, Maastricht, The Netherlands; 4 Vascular Surgery Department, University Hospital Aachen, Pauwelsstrasse 30, Aachen, 52074 Germany

**Keywords:** Carotid endarterectomy, Intraoperative neuromonitoring, Transcranial Doppler ultrasonography, Microembolic signals, Perioperative stroke, Carotid artery stenosis

## Abstract

**Purpose:**

This study aims to assess whether perioperative microembolization can identify patients at risk for postoperative stroke.

**Methods:**

A total of 257 carotid endarterectomy procedures in 254 patients were analyzed (154 symptomatic, 103 asymptomatic). Neuromonitoring, combining transcranial Doppler ultrasonography and continuous electroencephalography, was utilized to evaluate cerebral perfusion and detect microembolic signals (MES). Monitoring covered the entire intraoperative phase and a standardized 20-minute postoperative observation window.

**Results:**

Postoperative stroke occurred in seven patients, six of whom were symptomatic prior to the procedure. In exploratory penalized regression analyses, higher postoperative MES counts (OR 1.02 per MES, 95% CI 1.01–1.04; *p* < 0.001) and an intraoperative burden of ≥ 10 MES (OR 6.06, 95% CI 1.40–34.78; *p* = 0.016) were associated with postoperative stroke. Symptomatic patients exhibited a significantly higher embolic burden (6.7 ± 13.3 vs. 2.2 ± 5.0, *p* = 0.004).

**Conclusion:**

Beyond its role in guiding selective shunting, transcranial Doppler monitors the occurrence of microembolization, which could serve as a potential indicator of perioperative stroke. In this exploratory analysis, both an intraoperative burden of 10 or more MES and sustained postoperative microembolization were associated with postoperative stroke. Given the low number of events, these findings are hypothesis-generating and require prospective validation before they can guide perioperative management.

**Supplementary Information:**

The online version contains supplementary material available at https://doi.org/10.1007/s00423-026-04324-8.

## Introduction

Historically, carotid endarterectomy (CEA) has been established as the gold standard for stroke prevention in patients with high-grade symptomatic and asymptomatic stenosis, demonstrating its superiority over historical medical therapy in landmark trials such as NASCET and ACAS (Ferguson et al. 1999; Endarterectomy for asymptomatic carotid artery stenosis 1995; Halliday et al. 2010). However, with the advent of modern intensive medical management, the relative benefit of surgical revascularization is currently the subject of vivid debate, as reflected by trials such as CREST-2 and ECST-2 (Brott 2026; Donners et al. 2025). Consequently, for CEA to maintain its prognostic superiority today, it is more critical than ever to achieve ultra-low perioperative complication rates.

Although CEA remains an effective preventive strategy, the risk of a perioperative stroke remains the most critical complication of the procedure (Rao et al. 2020). Perioperative strokes primarily arise from two distinct mechanisms: thromboembolism, which accounts for the vast majority of events, and cerebral hypoperfusion during the mandatory phase of arterial cross-clamping (Riles et al. 1994). Consequently, an optimal intraoperative strategy must address both threats. To prevent hypoperfusion-related events, maintaining sufficient cerebral perfusion is essential. In current practice, a distinction is made between the routine and selective use of an intraluminal shunt (Squizzato et al. 2022). Because universal shunt placement is associated with specific procedural risks, such as vessel dissection, air embolism, and prolonged operative times, selective shunting based on intraoperative neuromonitoring (IONM) is increasingly favored (Naylor et al. 2023; Bond 2002; Kondov et al. 2023).

The current clinical practice guidelines of the European Society for Vascular Surgery (ESVS 2023) emphasize the necessity of precise intraoperative monitoring, particularly when the procedure is performed under general anesthesia (Naylor et al. 2023). In this context, electroencephalography (EEG) and transcranial Doppler (TCD) ultrasonography have proven to be highly effective complementary modalities (Jovanovic et al. 2023). While EEG detects changes in cortical neuronal activity resulting from ischemia, TCD allows not only for real-time measurement of hemodynamic flow velocities in the middle cerebral artery (MCA) but also the identification of microemboli, giving rise to microembolic signals (MES) in the TCD recording (Jahangiri et al. 2022).

However, the diagnostic value of these monitoring techniques depends heavily on the definition of clear alarm criteria and a standardized clinical algorithm.

The objective of this study was to evaluate the efficacy of continuous microembolic monitoring in identifying patients at risk for postoperative stroke. In particular, this study analyzes the impact of the perioperative microembolic burden on postoperative clinical outcomes, with a primary focus on neurological events such as stroke.

## Materials and methods

### Study design and patient population

**Fig. 1:**
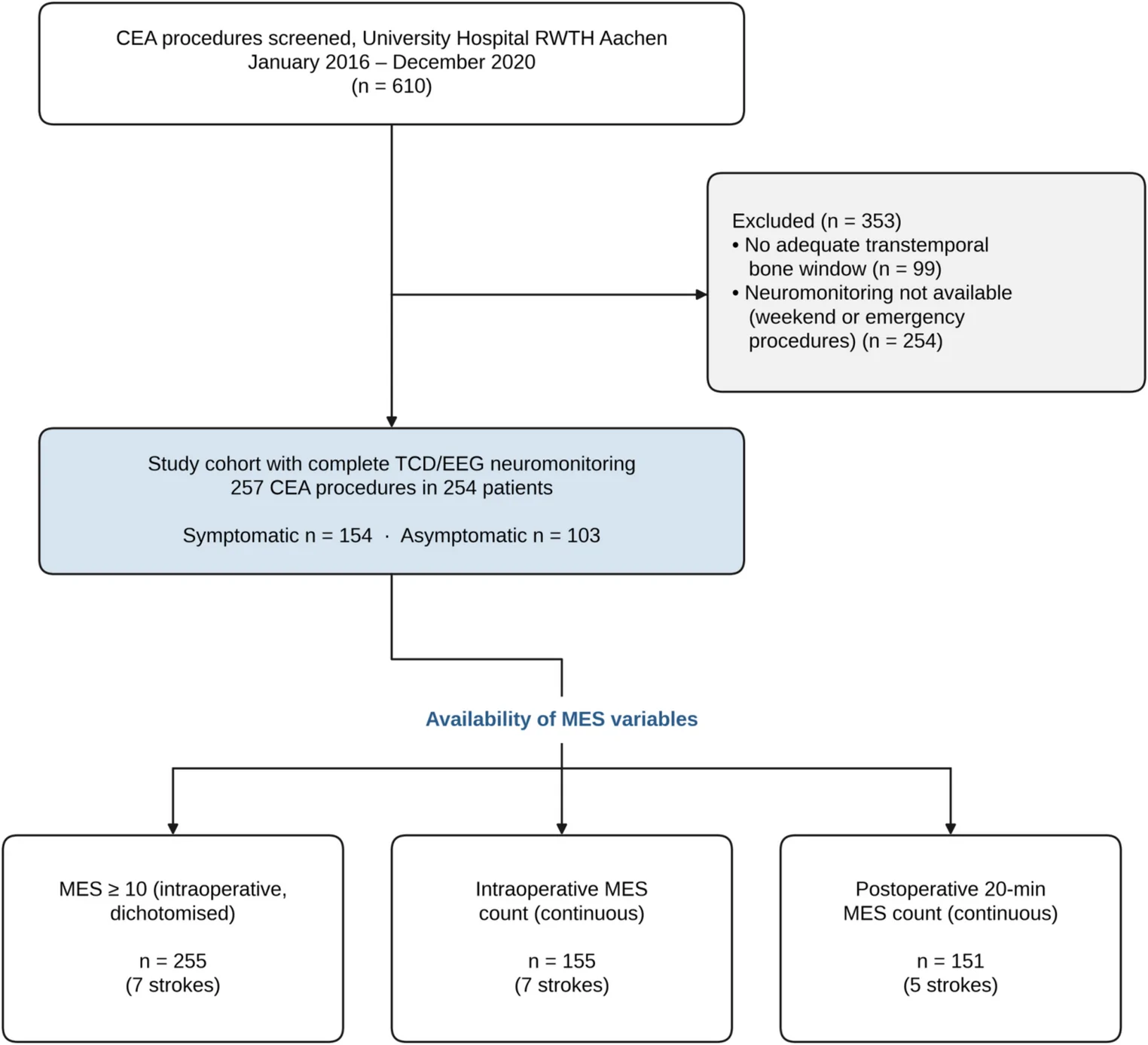
Flow chart of patient selection according to the STROBE statement. Screening was based on institutional procedure coding (January 2016 – July 2020) and manual review of operating room records (August – December 2020). The lower panel shows the number of procedures with available data for each microembolic signal (MES) variable. *CEA *carotid endarterectomy, *EEG *electroencephalography, *MES* microembolic signals, *TCD* transcranial Doppler

This single-center, retrospective observational study included 257 CEA procedures in 254 patients who underwent elective or urgent surgical revascularization via CEA between January 2016 and December 2020 at the University Hospital RWTH Aachen. The inclusion period was pre-defined to capture a stable retrospective cohort over a 5-year period. We limited the study inclusion period to December 2020 to ensure data homogeneity and to avoid the confounding effects introduced by the COVID-19 pandemic, which drastically altered elective surgery volumes and standard hospital lengths of stay. Importantly, our institutional neuromonitoring protocol and the routine intraoperative use of TCD for MES detection have not changed and remain our standard of care today. All adult patients undergoing elective or urgent CEA for high-grade carotid artery stenosis during the study period were screened using the institutional procedure coding records (January 2016 to July 2020) and a manual review of the operating room records (August to December 2020). Procedures were included if continuous intraoperative TCD and EEG neuromonitoring had been performed. Procedures were excluded if no adequate transtemporal bone window was present, or if neuromonitoring was not available because the procedure was performed on a weekend or as an emergency outside the regular availability of the neuromonitoring team (Fig. 1). CEA combined with cardiac surgery was not an exclusion criterion (*n*= 28). Three patients underwent two CEA procedures at different time points; the procedure was used as the unit of analysis, resulting in 257 procedures in 254 patients. The study was conducted in accordance with the ethical principles of the Declaration of Helsinki and was approved by the Ethics Committee of the Medical Faculty of RWTH Aachen University (EK 287/11). Due to the retrospective nature of the study and the use of anonymized clinical data, the requirement for individual informed consent for this analysis was waived by the Ethics Committee. Furthermore, this study was designed and reported in strict adherence to the Strengthening the Reporting of Observational Studies in Epidemiology (STROBE) guidelines. Baseline demographic data as well as the presence of cardiovascular risk factors and comorbidities were recorded. Perioperative risk was classified using the ASA (American Society of Anesthesiologists) physical status classification system. All patients underwent a standardized preoperative and postoperative clinical examination performed by a board-certified neurologist. Based on this clinical evaluation, patients were stratified into asymptomatic and symptomatic cohorts if an ipsilateral neurological deficit occurred within 6 months prior to presentation (Naylor et al. 2023).

### Preoperative diagnostic evaluation and workup

The primary diagnosis of carotid artery stenosis was established in all patients using duplex ultrasonography. The standardized preoperative workup additionally included cross-sectional imaging with either computed tomography angiography (CTA) or magnetic resonance angiography (MRA). All symptomatic patients additionally underwent cranial imaging (computed tomography or magnetic resonance imaging).

### Surgical procedure

Indications for surgery were determined on an interdisciplinary basis among neurologist, vascular surgeons and neuroradiologists in accordance with the clinical guidelines valid at the time of treatment (Naylor et al. 2018). All procedures were performed under general anesthesia. Surgical treatment involved standardized operative techniques, namely conventional endarterectomy with patch angioplasty, or eversion endarterectomy (EEA). The choice of surgical technique was primarily determined by anatomical considerations (e.g., EEA was preferred in cases of internal carotid artery elongation or kinking) as well as the attending surgeon’s preference.Systemic anticoagulation was achieved by administering 5000 IU of heparin prior to carotid artery clamping, and an activated clotting time (ACT) of ≥ 200 s was maintained throughout the procedure. Heparin was not routinely reversed with protamine at the conclusion of the procedure; its administration was strictly limited to cases where relevant surgical site bleeding was observed prior to wound closure.

### Intraoperative neuromonitoring and clinical algorithm

**Fig. 2:**
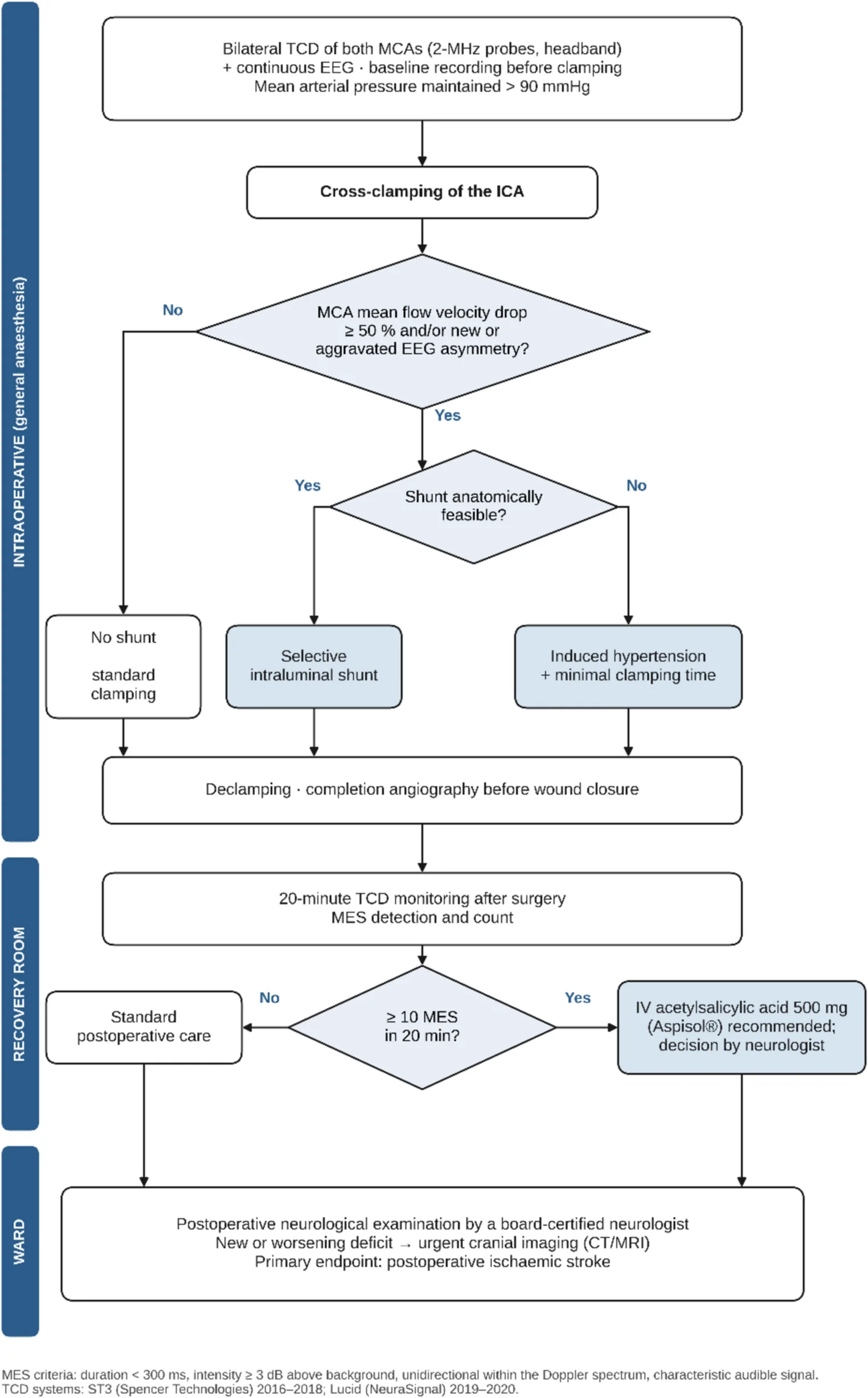
Institutional perioperative neuromonitoring algorithm. Bilateral transcranial Doppler (TCD) monitoring of both middle cerebral arteries (MCA) and continuous electroencephalography (EEG) were used to guide selective shunting during internal carotid artery (ICA) cross-clamping. Microembolic signals (MES) were detected automatically, with visual and acoustic verification by trained technicians, throughout surgery and during a standardized 20-minute postoperative window in the recovery room. MES criteria: duration < 300 ms, intensity ≥ 3 dB above background, unidirectional within the Doppler spectrum, characteristic audible signal. TCD systems: ST3 (Spencer Technologies) 2016–2018; Lucid (NeuraSignal) 2019–2020. *CT* computed tomography, *EEG* electroencephalography *ICA* internal carotid artery, *IV* intravenous, *MCA* middle cerebral artery, *MES *microembolic signals, *MRI* magnetic resonance imaging, *TCD* transcranial Doppler

The complete institutional neuromonitoring algorithm is summarized in Fig. 2. In all patients, mean arterial pressure was increased to > 90 mmHg to promote collateral cerebral perfusion to the cross-clamped hemisphere. Continuous intraoperative neuromonitoring was utilized to evaluate cerebral perfusion and detect microemboli during the procedure. This consisted of continuous EEG combined with TCD ultrasonography, provided an adequate temporal bone window was present. Two 2-MHz transducers were mounted over the temporal bone on each side using an adjustable headband. Until the end of 2018, the ST3 system (Spencer Technologies, Seattle, WA, USA) was used; from 2019 onwards, the Lucid system (NeuraSignal, Los Angeles, CA, USA) was used, with identical transducers, headband fixation, monitoring protocol and personnel. Baseline values were recorded before carotid cross-clamping. The setup, continuous real-time monitoring, and advanced interpretation of both the EEG and TCD modalities were performed by the same team of experienced neuro-Doppler technicians, supervised by a board-certified neurologist, throughout the procedure and the postoperative monitoring period. While bilateral monitoring was routinely attempted, insonation focused on the ipsilateral MCA when anatomically challenging. The indication for selective intraluminal shunt placement was based on predefined electrophysiological and hemodynamic alarm criteria (Vuurberg et al. 2023). A relevant decrease in cerebral perfusion was defined as an initial, post-clamp relative drop of the mean blood flow velocity in the MCA on TCD by ≥ 50% and/or significant EEG changes, characterized by the appearance of a new cerebral asymmetry or the aggravation of a pre-existing asymmetry compared to baseline values prior to clamping (Aburahma et al. 2011). If a patient reached or exceeded these critical thresholds, and shunt placement was anatomically feasible, a shunt was inserted to maintain adequate cerebral oxygenation. The anatomical feasibility of shunt placement was initially evaluated based on preoperative imaging and subsequently confirmed intraoperatively by the attending vascular surgeon following vessel exposure. In cases where shunt insertion was deemed anatomically impossible despite neuromonitoring signs of insufficient perfusion, an interdisciplinary strategy was applied: collateral cerebral blood flow was maximized by pharmacologically inducing systemic hypertension in close coordination with the anesthesiologist, and the carotid cross-clamping time was kept to the absolute minimum required for safe plaque removal. In cases of sufficient collateral perfusion—indicated by the absence of the aforementioned hemodynamic or electrophysiological alarm criteria—the procedure was continued without shunt protection. In our routine workflow, standard cross-clamping times were dictated by the time required for meticulous plaque removal and precise patch suturing to prevent restenosis and emboli. The strategy of expediting the procedure to keep the clamping time to an ‘absolute minimum’ was reserved for cases of intraoperative ischemia where shunt placement was not feasible. Completion angiography was performed at the end of each procedure before wound closure.

### Detection of microembolic signals

MES were detected continuously throughout all operative phases (dissection, cross-clamping, shunting and declamping) using the automated embolus-detection function of the respective TCD system. All signals were additionally verified in real time by the technicians, who monitored the Doppler spectrum visually and acoustically via headphones in order to distinguish true MES from artifacts (e.g., probe movement or electrocautery). MES were defined according to international consensus criteria as transient (< 300 ms), high-intensity signals (≥ 3 dB above the background spectrum), unidirectional within the Doppler spectrum and accompanied by a characteristic audible signal (Bernd Ringelstein et al. 1998). Three MES variables were analyzed: (i) the cumulative number of intraoperative MES per procedure (continuous variable); (ii) a high intraoperative embolic burden, defined as ≥ 10 intraoperative MES (dichotomized variable); and (iii) the number of MES during the 20-minute postoperative monitoring window (continuous variable). Intraoperative MES counts were documented but did not trigger changes to the surgical procedure.

### Postoperative monitoring

After extubation, TCD monitoring was continued for a standardized 20-minute period in the recovery room, with MES detection and verification performed in the same manner as intraoperatively. If ≥ 10 MES were detected during this window, intravenous acetylsalicylic acid (Aspisol^®^, 500 mg) was recommended; the final decision was made by the supervising neurologist. No further standardized measures were triggered by MES findings. All patients subsequently underwent a postoperative neurological examination by a board-certified neurologist, as described below.

### Clinical endpoints and postoperative monitoring

The primary clinical endpoint was the occurrence of a postoperative ischemic stroke, defined in accordance with current clinical guidelines as a new focal or global neurological deficit (or worsening of a pre-existing deficit) after an uneventful recovery from anesthesia, with symptoms lasting > 24 h (Naylor et al. 2023). To ensure accurate assessment of this endpoint, all patients underwent a postoperative examination by a neurologist. If the postoperative neurological examination revealed any new or worsening neurological deficit, urgent cranial imaging (computed tomography or magnetic resonance imaging) was immediately performed to confirm the diagnosis of an ischemic stroke.

Secondary objectives included surgical and system-related complications. These comprised postoperative bleeding/hematomas requiring surgical revision and iatrogenic lesions of the lower cranial nerves (hypoglossal nerve, vagus nerve), which presented clinically as dysphagia, among other symptoms.

### Statistical analysis

Basic statistical analyses were performed using SPSS (Version 29.0, IBM Corp., Armonk, NY, USA). Categorical variables were presented as absolute and relative frequencies and compared using Fisher’s exact test. Continuous variables were expressed as means with standard deviations and compared using Welch’s t-test.

To evaluate the discriminative ability of the intraoperative microembolic burden for postoperative stroke, a receiver operating characteristic (ROC) curve analysis was performed in patients with a documented absolute MES count (*n* = 155). The 95% confidence interval of the area under the curve (AUC) was calculated using DeLong’s method, and the cut-off was determined using the Youden index. Because this threshold was derived and tested in the same dataset, it is considered exploratory.

Given the low number of postoperative strokes (*n* = 7), the multivariable analysis was designed a priori as exploratory and hypothesis-generating, and the number of variables per model was deliberately restricted to avoid overfitting. Microembolic burden was the exposure of interest. Symptomatic status was selected a priori as the only clinical covariate, as it is the most established clinical risk factor for perioperative stroke after CEA and was associated with embolic burden in our cohort; variable selection was therefore not based on univariable significance. Logistic regression with Firth’s penalized likelihood was used to reduce small-sample and sparse-data bias; 95% confidence intervals (CIs) and p-values were derived from the profile penalized likelihood. All models were fitted as complete-case analyses.

Two models were constructed. The continuous model included symptomatic status, the cumulative intraoperative MES count and the MES count during the 20-minute postoperative window. The threshold model included symptomatic status and the dichotomized variable ≥ 10 intraoperative MES. To keep the number of variables per event as low as possible, the dichotomized and continuous MES variables were analyzed in separate models.

To assess the robustness of the associations between MES and stroke, sensitivity analyses were performed in which each of the following potential confounders was added individually to the respective model: age, sex, ASA class III–IV, diabetes mellitus, selective shunting, surgical technique (eversion vs. conventional endarterectomy with patch angioplasty), a post-clamp flow velocity drop ≥ 50%, and, for the postoperative model, postoperative intravenous acetylsalicylic acid. Given the exploratory design, no adjustment for multiple testing was performed, and p-values of descriptive group comparisons should be interpreted accordingly. Odds ratios (ORs) with 95% CIs are reported. Penalized regression analyses were performed using the R package logistf (R Foundation for Statistical Computing, Vienna, Austria), and a two-sided p-value < 0.05 was considered statistically significant.

## Results

### Baseline patient characteristics and demographics

Between January 2016 and December 2020, 610 CEA procedures were screened. After exclusion of 353 procedures (no adequate transtemporal bone window, *n* = 99; neuromonitoring not available because of weekend or emergency surgery, *n* = 254), 257 CEA procedures in 254 patients were included (Fig. 1). The study cohort had a mean age of 70.1 ± 9.8 years, and the majority of patients were male (77.8%, *n* = 200). The mean BMI was 27.3 ± 4.1 kg/m².

Analysis of the cardiovascular risk profile revealed a high prevalence of comorbidities: 92.2% (*n* = 237) of the patients presented with arterial hypertension, 35.8% (*n* = 92) suffered from diabetes mellitus, and nearly half of the cohort (48.6%, *n* = 125) were current or previous smokers. Atrial fibrillation was present in 18.3% (*n* = 47) of cases.

Regarding the preoperative neurological status, 59.9% (*n* = 154) of the surgical indications were based on symptomatic stenosis. Within this symptomatic subgroup, the predominant index events were a prior stroke (*n* = 96, 62.3% of symptomatic cases) or a transient ischemic attack (*n* = 41, 26.6%). The remaining 40.1% (*n* = 103) of patients underwent CEA for asymptomatic lesions. Of the 257 procedures, 98 (38.1%) were conventional endarterectomies with patchplasty and 155 (60.3%) were eversion endarterectomies and 4 (1.6%) were reconstructions with an interposition graft. Baseline demographics and comorbidities did not differ significantly between patients who developed a postoperative stroke and those who did not. Details of the demographic characteristics of the studied cohort are found in Table 1.

**Table 1:** Baseline demographic and clinical characteristics of the study cohort (*n* = 257 procedures), stratified by postoperative stroke occurrence

Parameter	Overall (*n* = 257)	No Stroke (*n* = 250)	Stroke (*n* = 7)	*p*-value*
Age (years), Mean ± SD	70.1 ± 9.8	70.3 ± 9.8	63.4 ± 8.8	0.086
Male sex, *n* (%)	200 (77.8%)	196 (78.4%)	4 (57.1%)	0.185
BMI (kg/m²), Mean ± SD	27.3 ± 4.1	27.2 ± 4.1	28.7 ± 4.4	0.403
Atrial fibrillation (AFib), *n* (%)	47 (18.3%)	47 (18.8%)	0 (0.0%)	0.356
Arterial hypertension, n (%)	237 (92.2%)	230 (92.0%)	7 (100.0%)	1
Malignancy, *n* (%)	40 (15.6%)	39 (15.6%)	1 (14.3%)	1
- Active malignancy, *n* (%)	12 (4.7%)	12 (4.8%)	0 (0.0%)	1
- History of malignancy, *n* (%)	28 (10.9%)	27 (10.8%)	1 (14.3%)	0.559
Diabetes mellitus, *n* (%)	92 (35.8%)	88 (35.2%)	4 (57.1%)	0.253
Smoking status, *n* (%)	125 (48.6%)	120 (48.0%)	5 (71.4%)	0.271
- Active smoking, *n* (%)	88 (34.2%)	85 (34.0%)	3 (42.9%)	0.694
- History of smoking, *n* (%)	37 (14.4%)	35 (14.0%)	2 (28.6%)	0.266
ASA score, Mean ± SD	2.9 ± 0.6	2.9 ± 0.6	3.0 ± 0.9	0.885
Symptomatic status, *n* (%)	154 (59.9%)	148 (59.2%)	6 (85.7%)	0.248
- Stroke, *n* (%)	96 (37.4%)	93 (37.2%)	3 (42.9%)	0.715
- TIA, *n* (%)	41 (16.0%)	38 (15.2%)	3 (42.9%)	0.083
- Amaurosis fugax, *n* (%)	11 (4.3%)	11 (4.4%)	0 (0.0%)	1
- Amaurosis fugax & TIA, *n* (%)	6 (2.3%)	6 (2.4%)	0 (0.0%)	1
Preoperative Aspirin, *n* (%)	171 (66.5%)	166 (66.4%)	5 (71.4%)	1
Preoperative Clopidogrel, *n* (%)	29 (11.3%)	29 (11.6%)	0 (0.0%)	1
Preoperative anticoagulation, *n* (%)	27 (10.5%)	27 (10.8%)	0 (0.0%)	1

Values are presented as mean ±standard deviation for continuous variables and as absolute numbers with percentages *n* (%) for categorical variables. * *p*-values were determined using appropriate tests. A *p*-value of < 0.05 was considered statistically significant

*ASA* American Society of Anesthesiologists, *BMI * body mass index, *SD* standard deviation, *TIA *transient ischemic attack

### Intraoperative parameters and the impact of symptomatic status

Immediately following cross-clamping of the internal carotid artery, the mean initial drop in TCD flow velocity was 38.8 ± 26.2%. Symptomatic patients demonstrated a significantly more pronounced initial perfusion drop compared to asymptomatic patients (42.1 ± 27.1% vs. 34.0 ± 24.1%, *p* = 0.015). According to the predefined protocol, 72 patients (29.4%) reached the critical thresholds for hypoperfusion, of whom 45 received a selective intraluminal shunt. The remaining 27 patients were managed without a shunt, with induced hypertension and minimized clamping time (mean clamping time 32.7 ± 12.7 min); one of these patients experienced a postoperative stroke. Overall, a shunt was placed in 20.2% (*n* = 52) of procedures. The shunting rate was higher in symptomatic patients (23.4%) compared to asymptomatic patients (15.5%), though this difference did not reach statistical significance (*p* = 0.154). For patients managed without a shunt, the mean absolute clamping time was 31.2 ± 12.2 min.

Real-time microemboli detection via TCD revealed that symptomatic patients exhibited a significantly higher intraoperative embolic load compared to the asymptomatic cohort. They demonstrated a higher overall MES detection rate (77.9% vs. 59.8%, *p* = 0.002) and a greater absolute mean number of intraoperative MES (6.7 ± 13.3 vs. 2.2 ± 5.0, *p* = 0.004). (For detailed intraoperative parameters, see Table 2).

**Table 2:** Intraoperative and early postoperative neuromonitoring parameters stratified by preoperative symptomatic status and by postoperative stroke occurrence

		Preoperative status			Postoperative stroke		
Parameter	Overall (*n* = 257)	Asymptomatic (*n* = 103)	Symptomatic (*n* = 154)	*p*	No stroke (*n* = 250)	Stroke (*n* = 7)	*p*
Intraoperative MES detected, *n* (%)	181 (70.7%)	61 (59.8%)	120 (77.9%)	0.002	175 (70.3%)	6 (85.7%)	0.677
Number of intraoperative MES, mean ± SD	4.8 ± 10.9	2.2 ± 5.0	6.7 ± 13.3	0.004	4.4 ± 10.6	14.0 ± 12.6	0.090
≥ 10 intraoperative MES, *n* (%)	64 (25.1%)	17 (16.8%)	47 (30.5%)	0.018	59 (23.8%)	5 (71.4%)	0.012
Initial MCA flow velocity drop after clamping (%), mean ± SD	38.8 ± 26.2	34.0 ± 24.1	42.1 ± 27.1	0.015	38.6 ± 26.3	45.4 ± 24.0	0.485
MCA flow velocity drop ≥ 50%, *n* (%)	72 (29.4%)	23 (23.2%)	49 (33.6%)	0.088	69 (29.0%)	3 (42.9%)	0.422
Selective shunt placement, *n* (%)	52 (20.2%)	16 (15.5%)	36 (23.4%)	0.154	49 (19.6%)	3 (42.9%)	0.149
Clamping time without shunt (min), mean ± SD	31.2 ± 12.2	29.7 ± 11.8	32.2 ± 12.5	0.158	31.0 ± 12.2	38.5 ± 13.3	0.342
Postoperative MES within 20 min, mean ± SD	15.5 ± 41.0	12.1 ± 29.2	17.9 ± 47.7	0.356	10.3 ± 24.7	168.2 ± 104.3	0.028
Postoperative IV acetylsalicylic acid, *n* (%)	18 (7.0%)	6 (5.8%)	12 (7.8%)	0.625	14 (5.6%)	4 (57.1%)	< 0.001

Values are presented as mean ± standard deviation or as n (%). Categorical variables were compared using Fisher’s exact test and continuous variables using Welch’s t-test; *p* < 0.05 was considered statistically significant. Percentages and means refer to procedures with available data: intraoperative MES detection, *n* = 256; number of intraoperative MES, *n* = 155; ≥10 intraoperative MES, *n* = 255; MCA flow velocity drop, *n* = 245; clamping time without shunt, *n* = 197; postoperative MES within 20 min, *n* = 151 (5 strokes)

*IV* intravenous, *MCA* middle cerebral artery* MES *microembolic signals, *SD* standard deviation

### Microembolic burden and postoperative stroke

**Fig. 3:**
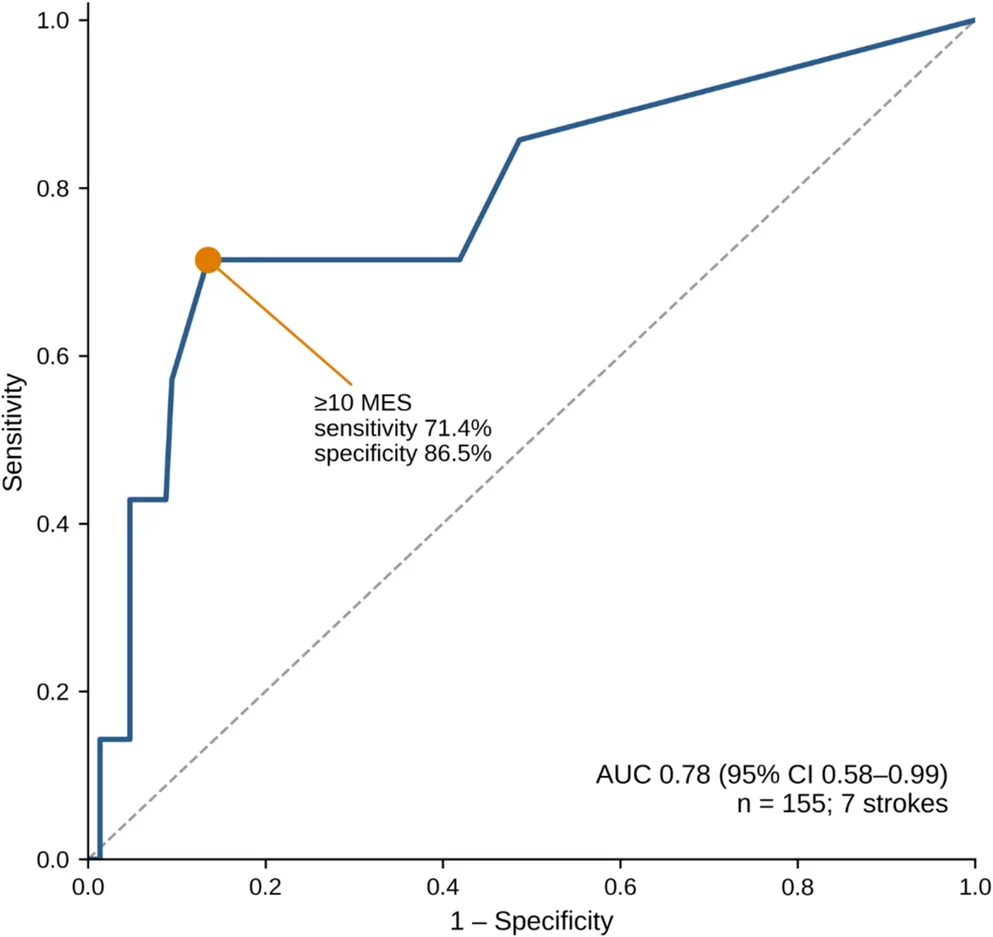
Receiver operating characteristic (ROC) curve evaluating the predictive accuracy of intraoperative microembolic signals (MES) for postoperative stroke (*n* = 155 procedures with a documented MES count; 7 strokes). The area under the curve (AUC) is 0.78 (95% CI 0.58–0.99), with an optimal cut-off threshold of ≥ 10≥ 10ES achieving a sensitivity of 71.4% and a specificity of 86.5% (highlighted by the orange marker). ROC, receiver operating characteristic; AUC, area under the curve; MES, microembolic signal

The occurrence of a postoperative stroke was recorded in seven patients (2.7%), six of whom were symptomatic prior to the procedure. All six presented as a clinical worsening of pre-existing deficits. An exploratory evaluation of intraoperative parameters indicated an association between microembolic burden and adverse neurological outcomes. In procedures with a documented MES count (*n* = 155; 7 strokes), an exploratory ROC analysis yielded an AUC of 0.78 (95% CI 0.58–0.99), with a Youden index-derived threshold of ≥ 10 MES showing a sensitivity of 71.4% and a specificity of 86.5% (Fig. 3). Applied to all procedures with the dichotomized variable (*n* = 255), ≥ 10 intraoperative MES were observed in 71.4% (*n* = 5) of patients who experienced a postoperative stroke, compared to 23.8% (*n* = 59) in the non-stroke group (*p* = 0.012). Furthermore, symptomatic patients more frequently exceeded this threshold than the asymptomatic cohort (30.5% vs. 16.8%, *p* = 0.018). Given the descriptive design and limited event rate, these findings remain strictly hypothesis-generating and suggest that intraoperative MES counts may serve as an exploratory risk indicator rather than a definitive predictor, warranting further prospective validation. Other intraoperative parameters were not significantly associated with stroke. While the mean absolute number of intraoperative MES was numerically higher in the stroke group (14.0 ± 12.6 vs. 4.4 ± 10.6), this difference was not statistically significant (*p* = 0.090). Similarly, the mere presence of any MES (85.7% vs. 70.3%, *p* = 0.677) and the mean initial TCD flow velocity drop (45.4% vs. 38.6%, *p* = 0.485) did not differ significantly between groups. Although the rate of selective shunt insertion was notably higher in the stroke group compared to the non-stroke cohort (42.9% vs. 19.6%), this difference did not reach statistical significance (*p* = 0.149). However, the quantitative analysis of postoperative embolism within a 20-minute window following reperfusion showed an association with adverse outcomes; patients who developed a postoperative stroke exhibited a higher mean postoperative MES count compared to those without stroke (168.2 ± 104.3 vs. 10.3 ± 24.7, *p* = 0.028).

In the continuous model (complete-case analysis, *n* = 85; 5 events), the postoperative MES count was associated with stroke occurrence (OR 1.02 per MES, 95% CI 1.01–1.04; *p* < 0.001), whereas symptomatic status (OR 1.44, 95% CI 0.13–24.43; *p* = 0.763) and the intraoperative MES count (OR 1.03, 95% CI 0.95–1.07; *p* = 0.249) were not (Table 2).

In the exploratory threshold model (*n* = 255; 7 events), ≥ 10 intraoperative MES was associated with stroke occurrence (OR 6.06, 95% CI 1.40–34.78; *p* = 0.016), whereas symptomatic status was not (OR 2.16, 95% CI 0.42–21.37; *p* = 0.377). In sensitivity analyses in which age, sex, ASA class, diabetes mellitus, selective shunting, surgical technique, a flow velocity drop ≥ 50% or postoperative intravenous acetylsalicylic acid were added individually, the direction and magnitude of both associations remained largely unchanged (≥ 10 MES: OR range 4.86–7.44; postoperative MES count: OR range 1.020–1.029 per MES; Supplementary Table S1). Given the small number of events, all estimates are imprecise and should be regarded as hypothesis-generating. The full regression results are shown in Table 3; further details regarding intraoperative parameters can be found in Table 2.

**Table 3:** Firth penalized logistic regression models for postoperative stroke

Variable	Univariable OR (95% CI)	*p*-value	Multivariable OR (95% CI)	*p*-value
Continuous model (multivariable: *n* = 85; 5 events)				
Symptomatic status	2.99 (0.62–29.01)	0.187	1.44 (0.13–24.43)	0.763
Intraoperative MES count, per MES	1.03 (1.00–1.07)	0.050	1.03 (0.95–1.07)	0.249
Postoperative 20-min MES count, per MES	1.03 (1.02–1.04)	< 0.001	1.02 (1.01–1.04)	< 0.001
Threshold model (multivariable: *n* = 255; 7 events)				
Symptomatic status	2.99 (0.62–29.01)	0.187	2.16 (0.42–21.37)	0.377
≥ 10 intraoperative MES	7.01 (1.64–39.80)	0.009	6.06 (1.40–34.78)	0.016

Firth’s penalized likelihood logistic regression; 95% CIs and p-values derived from the profile penalized likelihood; complete-case analyses. Univariable models contain only the respective variable (symptomatic status, *n* = 257; ≥10 intraoperative MES, *n* = 255; intraoperative MES count, *n* = 155; postoperative MES count, *n* = 151). Absolute MES counts were available in 155 (intraoperative) and 151 (postoperative) patients, and the dichotomized variable ≥ 10 intraoperative MES in 255 patients; two of the seven stroke patients had no postoperative MES recording

*CI* confidence interval, *MES* microembolic signals, *OR* odds ratio

### Postoperative complications and clinical outcomes

Within the in-hospital observation period, the primary endpoint of postoperative stroke occurred in 1.0% (*n* = 1) of the asymptomatic cohort and 3.9% (*n* = 6) of the symptomatic cohort (*p* = 0.248). Overall rates of surgical site complications, systemic adverse events, and non-neurological mortality (1.2%) were low. The mean length of hospital stay was 7.4 ± 5.0 days. Reflecting their higher perioperative morbidity, symptomatic patients required a significantly longer hospitalization compared to asymptomatic patients (9.2 ± 5.5 vs. 4.7 ± 1.9 days, *p* < 0.001).

The occurrence of a new perioperative stroke severely impacted the postoperative clinical course. Patients who suffered a stroke experienced a prolonged hospital stay compared to the complication-free cohort (16.1 vs. 7.2 days, *p* = 0.002) and a profound shift in discharge disposition. Instead of returning home, the majority of stroke patients required transfer to a rehabilitation facility (57.1% vs. 7.2%, *p* = 0.001). A comprehensive overview of postoperative outcomes, length of stay, and discharge dispositions is provided in Table 4.

**Table 4:** Postoperative outcomes and complications stratified by preoperative symptomatic status and by postoperative stroke occurrence

		Preoperative status			Postoperative stroke		
Parameter	Overall (*n* = 257)	Asymptomatic (*n* = 103)	Symptomatic (*n* = 154)	*p*	No stroke (*n* = 250)	Stroke (*n* = 7)	*p*
Postoperative stroke, *n* (%)	7 (2.7%)	1 (1.0%)	6 (3.9%)	0.248	–	–	–
Cranial nerve injury, *n* (%)	8 (3.1%)	3 (2.9%)	5 (3.2%)	1.000	8 (3.2%)	0 (0.0%)	1.000
Dysphagia, *n* (%)	8 (3.1%)	2 (1.9%)	6 (3.9%)	0.482	7 (2.8%)	1 (14.3%)	0.201
Postoperative hematoma requiring revision, *n* (%)	6 (2.3%)	4 (3.9%)	2 (1.3%)	0.222	6 (2.4%)	0 (0.0%)	1.000
Wound healing disorder, *n* (%)	2 (0.8%)	0 (0.0%)	2 (1.3%)	0.518	2 (0.8%)	0 (0.0%)	1.000
STEMI, *n* (%)	1 (0.4%)	0 (0.0%)	1 (0.6%)	1.000	1 (0.4%)	0 (0.0%)	1.000
NSTEMI, *n *(%)	1 (0.4%)	0 (0.0%)	1 (0.6%)	1.000	0 (0.0%)	1 (14.3%)	0.027
Postoperative pneumonia, *n *(%)	8 (3.1%)	0 (0.0%)	8 (5.2%)	0.023	8 (3.2%)	0 (0.0%)	1.000
Length of hospital stay (days), mean ± SD	7.4 ± 5.0	4.7 ± 1.9	9.2 ± 5.5	< 0.001	7.2 ± 4.7	16.1 ± 4.6	0.002
Persistence of symptoms at discharge, *n *(%)	64 (24.9%)	1 (1.0%)	63 (40.9%)	< 0.001	n/a†	n/a†	–
Discharged home, *n *(%)	174 (67.7%)	71 (68.9%)	103 (66.9%)	0.786	173 (69.2%)	1 (14.3%)	0.005
Discharged to rehabilitation, *n *(%)	22 (8.6%)	1 (1.0%)	21 (13.6%)	< 0.001	18 (7.2%)	4 (57.1%)	0.001
Transferred to another hospital, *n *(%)	18 (7.0%)	1 (1.0%)	17 (11.0%)	0.002	16 (6.4%)	2 (28.6%)	0.079
Death (other causes), *n *(%)	3 (1.2%)	1 (1.0%)	2 (1.3%)	1.000	3 (1.2%)	0 (0.0%)	1.000

Values are presented as mean ± standard deviation or as n (%). Categorical variables were compared using Fisher’s exact test and continuous variables using Welch’s t-test; *p* < 0.05 was considered statistically significant. Length of stay refers to the stay on the vascular surgery ward. †Not applicable stratified by stroke occurrence

*NSTEMI* non-ST-elevation myocardial infarction, *SD* standard deviation *STEMI* ST-elevation myocardial infarction

## Discussion

While our center routinely employs a multimodal neuromonitoring approach (combining TCD and EEG) to e.g. guide a safe selective shunting strategy, this study focuses specifically on the unique capability of continuous TCD monitoring to detect and quantify cerebral microembolization (Kondov et al. 2023; Finocchi et al. 1997). Our results suggest that this real-time quantification may provide relevant perioperative information, as the microembolic burden during both the intraoperative and the postoperative phase was associated with adverse neurological outcomes. During the procedure, a high embolic burden exceeding the critical threshold of ≥ 10 MES was associated with a higher rate of postoperative stroke. Following reperfusion, higher postoperative MES counts during the first 20 postoperative minutes were associated with postoperative stroke, suggesting that the cumulative embolic burden rather than isolated signals is of clinical relevance.

As described in the systematic review byUdesh et al., the vast majority of perioperative strokes are embolic in origin, with only about 20 % being attributable to hemodynamic changes during carotid cross-clamping (Udesh et al. 2017). This predominantly embolic etiology underscores the critical role of TCD, as it remains the sole intraoperative monitoring modality capable of detecting and quantifying real-time microembolization. In our cohort, while the mere presence of any MES was common and did not correlate with postoperative stroke occurrence, absolute MES counts were numerically higher in the stroke group. In our exploratory ROC-based threshold analysis, the occurrence of ≥ 10 intraoperative MES was associated with postoperative stroke; this critical embolic threshold was reached by 71.4% of stroke patients compared to 23.8% of the non-stroke group (*p* = 0.012).

Symptomatic patients in our cohort carried a significantly higher embolic burden, which is likely due to plaque morphology. Kopczak et al. found that symptomatic carotid disease was strongly associated with highly vulnerable, complicated plaques, which are known to be intrinsically linked to a higher rate of cerebral microembolization (Kopczak et al. 2022; Altaf et al. 2014). Consequently, such friable plaques are markedly more susceptible to mechanical fragmentation during surgical manipulation, explaining the elevated intraoperative embolic load observed in the symptomatic subgroup of our study. This reinforces the clinical imperative to employ precise surgical techniques and a strict ‘minimal touch’ approach during vessel dissection, particularly in symptomatic cases.

Persistent MES after completion of the reconstruction and removal of the emboligenic plaque may reflect thrombus formation or platelet aggregation at the endarterectomy surface, the arteriotomy suture line, intimal flaps, residual plaque irregularities, or turbulence-related activation of the coagulation cascade. In contrast to emboli released during dissection or clamping, postoperative embolic signals may therefore represent an ongoing and potentially modifiable embolic source. Continuous surveillance in the early postoperative phase is essential to identify patients experiencing these embolic clusters. Despite these associations, the critical question remains how these findings translate into clinical routine for the vascular surgeon. Currently, intraoperative MES detection serves primarily as a real-time risk stratification tool rather than a definitive mandate for standardized protocol changes. In our institutional protocol, the only MES-triggered measure was the recommendation of intravenous ASA in patients with ≥ 10 MES during the postoperative monitoring window; intraoperative MES counts were documented without procedural consequences. The detection of high embolic activity acts as an immediate early warning system. Intraoperatively, a sudden burst of MES may prompt immediate technical re-evaluation, such as rigorous flushing, adjustment of clamp release techniques, or a critical review of the completion angiography to rule out minor intimal flaps or suture line irregularities. Furthermore, if high embolic activity persists into the early postoperative phase, supported by historical evidence and consensus guidelines, targeted pharmacological interventions (e.g., the administration of acetylsalicylic acid [ASA]) or even prompt surgical re-exploration may be considered to prevent permanent neurological damage (Naylor et al. 2023; Hayes et al. 2000). Postoperatively, the overall MES burden can guide clinical triaging: patients exceeding the high-risk threshold may benefit from intensified neurological observation and stricter hemodynamic management. Nevertheless, we emphasize that given the overall low number of stroke events, our findings remain observational and hypothesis-generating. Until prospective trials can validate specific actionable interventions, these data should be interpreted as an individualized patient safety tool rather than a strict guideline for altering established surgical routines.

Routine versus selective shunt placement remains a core debate in carotid surgery. In our cohort, applying our protocol threshold of a ≥ 50% drop in TCD mean flow velocity led to selective shunt insertion in 20.2% of patients. While the absolute shunting rate did not differ significantly between groups, symptomatic patients exhibited a significantly more pronounced initial perfusion drop compared to asymptomatic patients (42.1% vs. 34.0%, *p*= 0.015). This confirms that symptomatic patients are more vulnerable to acute flow reductions due to an exhausted cerebrovascular reserve, reinforcing the value of continuous perfusion monitoring during cross-clamping (Gupta et al. 2012; Reinhard et al. 2003; Semenyutin et al. 2017).

While the absolute rate of postoperative stroke did not reach statistical significance between asymptomatic and symptomatic patients (1% vs. 3.9%, corresponding to 1vs. 6 events), the absolute and percentage differences indicate a clinically relevant trend toward a higher stroke rate in symptomatic patients.The higher baseline neurological and cardiopulmonary burden of this group translated into significantly prolonged hospital stays (9.2 vs. 4.7 days, *p* < 0.001) and a substantially higher need for specialized rehabilitation (13.6% vs. 1.0%, *p*< 0.001), aligning with contemporary literature that identifies symptomatic carotid disease as a surrogate for broader cardiopulmonary compromise (Mandelbaum et al. 2021; Zil-E-Ali et al. 2021).

Similarly, the occurrence of a perioperative stroke extended hospitalizations (16.1 vs. 7.2 days, *p* = 0.002) and shifted the majority of discharge dispositions toward rehabilitation facilities (57.1%). Ultimately, because these profound socio-economic and clinical burdens are primarily driven by perioperative ischemic events, optimizing patient outcomes requires multidisciplinary care and may benefit from neuromonitoring focused on the real-time detection and mitigation of cerebral microembolization.

In the current era of stroke prevention, the justification for CEA relies heavily on achieving ultra-low perioperative complication rates against the backdrop of considerably improved best medical treatment (BMT). The recent publication of the CREST-2 trials reported low annual stroke rates for asymptomatic patients under intensive medical therapy; however, as critically appraised by AbuRahma et al., such highly resourced, trial-level medical management is largely non-reproducible in real-world clinical practice (Brott 2026; AbuRahma et al. 2026). Consequently, the true real-world benefit of surgical revascularization could be even greater, and CEA continues to play an indispensable role in appropriately selected patients. Furthermore, contemporary trial data for symptomatic cohorts remains limited; for instance, while the recent ECST-2 interim analysis demonstrated the high baseline efficacy of modern optimized medical therapy, the trial included only 170 symptomatic patients, precluding definitive conclusions for this specific higher-risk subgroup (Donners et al. 2025).

Ultimately, the underlying imperative remains unchanged: to justify surgical intervention against increasingly effective medical therapy, minimizing procedural risk is paramount. Despite this pressing need to guarantee minimal perioperative morbidity, intraoperative neuromonitoring strategies remain highly heterogeneous across vascular centers, lacking universal standardization. Even when routine monitoring is applied, conventional intraoperative assessments—such as monitoring perfusion drops or electrical changes to guide selective shunting—capture only part of the risk profile. While these conventional tools are crucial for preventing immediate procedural ischemia, they fail to account for embolic complications. This is the critical gap our real-time transcranial Doppler findings aim to address. Our results suggest that systematically tracking intraoperative microembolization (particularly burdens exceeding the threshold of $\:\ge\:10$ MES) alongside extending real-time monitoring into the early postoperative period may provide additional prognostic information that is not captured by conventional hemodynamic monitoring.

Several limitations of this study should be acknowledged. First, the retrospective single-center design introduces an inherent risk of selection bias and limits the generalizability of the findings. Furthermore, due to this retrospective nature, standardized data on operative time were unavailable, and the results of the completion angiography were not archived in an analyzable form. Consequently, the technical quality of the arterial reconstruction—which directly impacts MES generation—remains an unmeasured confounder. Second, although the overall cohort was relatively large, the number of postoperative stroke events was low (*n* = 7), resulting in limited statistical power and wide confidence intervals in multivariable analyses. Consequently, the identified threshold of ≥ 10 intraoperative MES should be considered exploratory and requires external validation in larger prospective cohorts. In addition, absolute MES counts were not documented in all procedures (intraoperative, *n* = 155; postoperative, *n* = 151), and two of the seven stroke patients had no postoperative MES recording. Third, while TCD is occasionally noted for technical dependencies, it actually exhibits high interobserver agreement detecting MES compared to many other ancillary diagnostic modalities; nevertheless, study inclusion was inherently restricted to patients possessing an adequate temporal bone window. Furthermore, intraoperative TCD is susceptible to motion artifacts, probe displacement during surgical head repositioning or manipulation, and transient signal interference from electrical equipment like electrocautery, all of which require meticulous signal verification by trained personnel to accurately differentiate true microembolic signals from procedural artifacts. Moreover, the TCD system changed during the study period (ST3 until 2018, Lucid from 2019); although transducers, fixation, monitoring protocol and personnel remained unchanged and MES counts did not differ significantly between both periods, differences in the automated detection algorithms cannot be fully excluded. Fourth, although our postoperative surveillance was structured around a predefined early monitoring window, our institutional experience indicates that embolic activity clusters tightly within the immediate post-reconstruction phase, making truly delayed embolic clusters exceedingly rare if the initial observation period remains negative. Finally, while our analyses demonstrate an association between embolic burden and postoperative stroke, causality cannot be established, nor can residual confounding by plaque morphology, technical aspects of the procedure, or perioperative antithrombotic management, including the non-uniform administration of intravenous ASA in patients with postoperative microembolization, be excluded.

Despite these limitations, our study benefits from a standardized multimodal neuromonitoring protocol, a homogeneous surgical strategy, and detailed perioperative assessments of both hemodynamic and embolic parameters. Building upon the landmark neuromonitoring work by Ackerstaff et al., our findings are consistent with an association between cerebral microembolization and postoperative stroke in a contemporary cohort treated in the era of modern medical therapy (Ackerstaff et al. 2000). This study may therefore serve as a contemporary update on the prognostic relevance of TCD monitoring, which warrants prospective validation.

## Conclusion

Beyond its role in guiding selective shunting, transcranial Doppler monitors the occurrence of microembolization, which could serve as a potential indicator of perioperative stroke. In this exploratory analysis, both an intraoperative burden of 10 or more MES and sustained postoperative microembolization were associated with postoperative stroke. Given the low number of events, these findings are hypothesis-generating and require prospective validation before they can guide perioperative management. 

## Supplementary Information

Below is the link to the electronic supplementary material.

Supplementary Material 1 (DOCX 12.5 KB)

## Data Availability

No datasets were generated or analysed during the current study.
