# Supplementary material for: Neuromonitoring of perioperative microembolization and its association with postoperative stroke after carotid endarterectomy
Source: Langenbecks Arch Surg. 2026 Oct 9;411(1):272. doi: 10.1007/s00423-026-04324-8 (PMC13653865; doi:10.1007/s00423-026-04324-8)
Supplement: Supplementary file 1 [file 423_2026_4324_MOESM1_ESM.docx]

**Supplementary Table S1.** Sensitivity analyses: association of MES with postoperative stroke after individual addition of potential confounders.

| **Added covariate** | **≥10 intraoperative MES (n = 255; 7 events), OR (95% CI), p** | **Postoperative MES count, per MES (n = 151; 5 events), OR (95% CI), p** |
| --- | --- | --- |
| None (reference model) | 6.06 (1.40–34.78), 0.016 | 1.025 (1.014–1.041), <0.001 |
| Age (per 10 years) | 6.37 (1.45–36.84), 0.014 | 1.026 (1.014–1.049), <0.001 |
| Male sex | 6.53 (1.50–37.75), 0.012 | 1.022 (1.012–1.039), <0.001 |
| ASA class III–IV* | 4.86 (1.02–28.83), 0.047 | 1.024 (1.012–1.039), <0.001 |
| Diabetes mellitus | 5.72 (1.30–33.11), 0.021 | 1.029 (1.015–1.064), <0.001 |
| Selective shunting | 7.44 (1.66–44.22), 0.009 | 1.023 (1.013–1.039), <0.001 |
| Eversion endarterectomy | 6.51 (1.50–37.30), 0.012 | 1.024 (1.012–1.043), <0.001 |
| Flow velocity drop ≥50% | 6.36 (1.45–36.68), 0.014 | 1.024 (1.013–1.044), <0.001 |
| Postoperative IV acetylsalicylic acid | – | 1.020 (1.008–1.035), 0.001 |

Firth's penalized logistic regression. Each model contains symptomatic status, the respective MES variable and one additional covariate. The postoperative models do not include the intraoperative MES count, in order to use the larger complete-case sample (n = 151). *ASA class was missing in 17 patients (n = 238 with 6 events, and n = 144 with 4 events, respectively).

ASA, American Society of Anesthesiologists; CI, confidence interval; IV, intravenous; MES, microembolic signals; OR, odds ratio.
